# Typhus [ti′ fəs]

**DOI:** 10.3201/eid1506.999999

**Published:** 2009-06

From Greek τīϕος [*typhos*]*,* meaning heavy stupor; also related to Greek *typhein*, to smoke. A disease known since antiquity, typhus has been described as follows: “A kind of continued fever, attended with great prostration of the nervous and vascular systems, with a tendency to putrefaction in the fluids and vitiation in the secretions; putrid fever. A genus of the order *Febres*, class *Pyrexia*, of Cullen’s nosology” (J. Thomas, 1885).

Today, typhus refers to any of a group of acute infections caused by rickettsiae and transmitted to persons by the bite of arthropods such as fleas and lice. Epidemic typhus, caused by *Rickettsia prowazekii*, is characterized by headache, high fever, chills, rash, and, in serious cases, by stupor or lack of awareness of reality. Outbreaks usually occur in crowded or unsanitary environments.

